# Grain legume seed systems for smallholder farmers: Perspectives on successful innovations

**DOI:** 10.1177/0030727020953868

**Published:** 2020-09-01

**Authors:** Chris O Ojiewo, Lucky O Omoigui, Janila Pasupuleti, Jillian M Lenné

**Affiliations:** 1 28639 International Crops Research Institute for the Semi-Arid Tropics-Nairobi, Nairobi, Kenya; 2 105528 International Institute of Tropical Agriculture, Ibadan, Nigeria; 3 28639 International Crops Research Institute for the Semi-Arid Tropics-Patancheru, Hyderabad, Telangana, India; 4 North Oldmoss Croft, Fyvie, Turriff, Aberdeenshire, UK

**Keywords:** Grain legumes, integrated seed systems, market demand, seed policy environment, small seed packs, multistakeholder platforms

## Abstract

Grain legumes are nutritionally important components of smallholder farming systems in sub-Saharan Africa and Asia. Unfortunately, limited access to quality seed of improved varieties at affordable prices due to inadequate seed systems has reduced their contribution to improving nutrition and reducing poverty in these regions. This paper analyses four seed systems case studies: chickpea in Ethiopia and Myanmar; cowpea in Nigeria; and tropical grain legumes in Nigeria, Tanzania and Uganda highlighting outcomes, lessons learned, and the enabling factors which supported the successful innovations. All four case studies highlighted at least some of the following outcomes: increased adoption of improved varieties and area planted; increased productivity and income to farmers; improved market access and growth; and significant national economic benefits. Important lessons were learned including the value of small seed packets to reach many farmers; the value of innovative partnerships; capacity building of value chain actors; and continuity and coherence of funding through Tropical Legumes projects II and III and the recently funded Accelerated Varietal Improvement and Seed Delivery of Legumes and Cereals in Africa (AVISA) project. Successful adoption of innovations depends not just on the right technologies but also on the enabling environment. The case studies clearly showed that market demand was correctly identified, establishment of successful partners and institutional linkages overcame constraints in production and delivery of improved seed to smallholders, and fostered conducive policies supported national seed systems. All were integral to seed system viability and sustainability. It is hoped that these examples will provide potential models for future grain legume seed systems efforts. In addition, the analysis identified a number of areas that require further research.

## Grain legume seed systems in less-developed countries

Grain legumes are nutritionally important components of smallholder farming systems in much of sub-Saharan Africa and Asia. Their higher protein and micro-nutrient contents enhance diets and their ability to fix nitrogen improves soil health (Ojiewo et al., 2018). Good quality seeds are essential for productive legume crops. To enhance grain legume production in food production systems, the challenge of seed supply must be tackled to ensure availability, accessibility, affordability, and sustainability for smallholders. However recent studies have shown that only about 1% of grain legume seed used by smallholders is sourced from the formal sector (McGuire and Sperling, 2016).

Various factors contribute to lack of interest by the formal sector in grain legume seed production in less-developed countries. These include lower seed multiplication rate requiring extra space, labour and effort to move from breeder seed to certified/Quality Declared Seed (QDS) at commercial scale; relatively large seed size – for every hectare up to 100 kg of seed is needed for some legumes, which has cost implications for the storage and transportation of seed; and seeds of some legumes deteriorate rapidly after harvest, especially if shelled (Ojiewo et al., 2018). There are also challenges in the mechanization of some legume crops which hinders large-scale production. All these factors make producing and marketing grain legumes less attractive to seed companies and result in limited farmer access to quality and affordable seed of improved varieties.

Twenty years ago, a comprehensive study of national seed systems in Eastern and Southern Africa highlighted a number of strategic interventions necessary for the development of grain legume seed systems (Tripp, 2000). These included the need for public sector investment; simplification of regulatory procedures; improvements in seed systems policies; and the need for integration of and coherence in donor-funded seed systems development projects (Tripp, 2000). More recently, two reports have analysed in detail critical interventions for sustainable seed systems development in sub-Saharan Africa and provided examples of successful endeavours in a number of countries (Ojiewo et al., 2018; Sperling et al., 2017). In particular, drawing on experience from many attempts to establish grain legume seed systems, Ojiewo et al. (2018) proposed 12 principles for mainstreaming efficient legume seed systems in Eastern Africa (Box 1).

Box 1. Twelve principles for mainstreaming functional legume seed systems in Eastern Africa.

*Principle 1:* Mainstreaming legume seed systems requires a clear theory of change

*Principle 2:* Mainstreaming legume seed systems is supported by a robust policy environment and regional integration

*Principle 3:* Mainstreaming legume seed systems is supported by a strong institutional framework

*Principle 4:* Mainstreaming legume seed systems begins with tackling the early generation seed (EGS) supply challenge

*Principle 5:* Mainstreaming legume seed systems requires an integrated approach in tackling the commercial seed class challenge

*Principle 6:* Mainstreaming legume seed systems requires institutional capacity building

*Principle 7:* Mainstreaming legume seed systems is linked to the promotion of utilization for improving food and nutrition security

*Principle 8:* Mainstreaming legume seed systems involves market linkages and income security

*Principle 9:* Mainstreaming legume seed systems is linked to the role in agricultural and dietary diversification and resilience

*Principle 10:* Mainstreaming legume seed systems is linked to women and youth empowerment

*Principle 11:* Mainstreaming legume seed systems requires multi-stakeholder involvement

*Principle 12:* Mainstreaming legume seed systems requires well-defined seed and adoption roadmaps

From: Ojiewo et al. (2018)

Several good examples of the promotion of improved grain legume varieties and the establishment of viable seed systems in sub-Saharan Africa and Asia have been achieved by the CGIAR Research Program – Grain Legumes and Dryland Cereals (GLDC Annual Reports, 2018, 2019), its predecessors and its many partners. The main objective of this perspective paper is to qualitatively analyse four case studies – three in sub-Saharan Africa and one in Asia – highlighting the outcomes, lessons learned and, most importantly, the enabling factors which supported the successful innovations. It is hoped that these examples will provide potential models for future grain legume seed systems efforts.

## Case studies

### Chickpea in Ethiopia

Chickpea breeding efforts in Ethiopia started in the early-1970s, focusing mainly on yield improvement. Over the years, many challenges were identified necessitating concerted efforts in improvement for biotic (wilt root-rot complex, Ascochyta blight, podborer) and abiotic (heat, drought and frost) stress resistance/tolerance; farmer requirements (herbicide tolerance, machine-harvestability, early maturity, suitability for intercropping or double cropping) and market preferences (large-seeded, fast cooking); integrated crop management packages that include inputs such as efficient and effective rhizobia inoculants as well as efficient seed and technology delivery systems. Since 2007, the International Crops Research Institute for the Semi-Arid Tropics (ICRISAT) through the Tropical Legumes projects (TL II Phase I and Phase II, TL III) worked closely with partners in Ethiopia including the Ethiopian Institute for Agricultural Research (EIAR), Regional Agricultural Research Institutes, Higher Institutions of Learning, Regional and District Bureaus of Agriculture, Seed Enterprises, the International Centre for Agricultural Research in Dry Areas (ICARDA), Integrated Seed Sector Development (ISSD), the Feed the Future Chickpea Innovation Laboratory and the Alliance for a Green Revolution in Africa (AGRA) among others to address some of these challenges, contributing to the release of 10 improved varieties targeting various agro-ecologies of Ethiopia as well as the promotion of on-the-shelf varieties.

During 7 years, adoption of improved chickpea varieties rose from 31–80%; the share of chickpea growers rose from 65–90%; the area sown to chickpea per farm rose from 0.17–0.4 ha; and household welfare was significantly increased (GLDC Annual Report, 2018; Verkaart et al., 2019). Adoption was driven more by profits and disease tolerance than by higher yields (Michler et al., 2018; Verkaart et al., 2017). These efforts contributed to the average productivity of chickpea in Ethiopia increasing by nearly two-fold from an average of 1.27 t/ha in 2007 to 2.56 t/ha in 2019, generating US$61 million annually in export revenue (CSA, 2019). Investment in small seed packets for distribution to farmers raised awareness and farmers were more receptive to the value of improved varieties. A system was established for understanding end-user needs (both farmer and market) to inform the development of future improved varieties and a quality standards system was set up for both local and export grain. Scaling-up outputs and outcomes from TL II achieved more impact and built sustainable seed systems for chickpea in Ethiopia through TL III (Ojiewo et al., 2019).

The partnership between the EIAR, the extension services, ICRISAT and other value chain actors including farmers, seed producers, quality control actors and export grain aggregators laid the foundation for building the multi-stakeholder EthioPEA Alliance which was crucial in strengthening the chickpea value chain (Mekasha et al., 2020).

A seed production and distribution system was established through seed grower associations and more than 70,000 extension agents were involved in promoting, supporting and sustaining the chickpea seed system. Attracted by early successes, chickpea seed grower associations formed seed companies with additional support from AGRA and ISSD. Local markets for excess seed were accessible and demand from both local and export markets drove increased production. The Government of Ethiopia included chickpea in the 5-year agricultural growth strategy (Growth and Transformation Plan II 2015/16–2019/20) and in the Ethiopian Commodity Exchange (ECX) with direct investment from government budgets in crop improvement, seed systems and market development https://ethiopia.un.org/sites/default/files/2019-08/GTPII%20%20English%20Translation%20%20Final%20%20June%2021%202016.pdf).

### Chickpea in Myanmar

In Myanmar, chickpea is grown in the post-rainy season on residual moisture in Central Dry Zone (CDZ). Sagaing, Mandlay and Magway are the major chickpea growing divisions in the country. The Department of Agriculture Research (DAR) conducts research while the Department of Agriculture (DOA) is responsible for research and development activities on chickpea in collaboration with ICRISAT. During 1975 to 2017, more than 5200 ICRISAT breeding lines and gene bank accessions were shared with the DAR (unpublished data from ICRISAT). Several advanced breeding lines supplied by ICRISAT were found suitable for cultivation in the CDZ and led to release of eight improved Yezin cultivars.

The adoption of improved Yezin varieties was rapid during 2004–05, covering an area of 82% of the country (Than et al., 2007). Recent data from the DOA suggests that 92% of the chickpea area in 2018–19 was under the improved varieties developed through the ICRISAT-DAR partnership with Yezin 3 (ICRISAT Chickpea Variety – ICCV 2) covering 31% of the area followed by Yezin 6 (ICCV 92944) (17%), Yezin 4 (ICCV 88202) (13%) and Yezin 8 (ICCV 97314) (11%). These new chickpea varieties are high-yielding and mature early and have improved resistance to Fusarium wilt and good seed quality. The data from the DAR for 2017–18 indicates that in the Sagaing and Mandalay regions, the extra-early Kabuli variety Yezin 3 is the most popular while in the Magway region, the Desi variety Yezin 4 is the most popular.

Myanmar has witnessed a chickpea revolution in the last two decades. From 1998 to 2018, the chickpea area increased from 101,172 ha to 368,390 ha, and the yield doubled from 668 Kg/ha to 1384 Kg/ha resulting in a total production of 0.54 m tons in 2018 (FAOstat, 2018). During this period, chickpea transitioned from a subsistence crop to an export crop with an annual export value of US$30 million in 2017 (FAOstat, 2018). Adoption of improved varieties by the farmers in Myanmar contributed to increased yield and production of chickpea (Win et al., 2014) with over 160,000 farmers benefitting (GLDC Annual Report, 2019). The introduction and subsequent adoption of early maturing chickpea cultivars generated economic benefits estimated at US$152.8 million (GLDC Annual Report, 2019).

The partnership between ICRISAT and the DAR and the DoA lead to the establishment and development of 1343 Village Seed Banks in 495 villages of Central Dry Zone between 2015 and 2018 supported by the ACIAR funded MyPulses project (Kumara Charyulu et al., 2018). The extension service played an important role in creating awareness of improved chickpea varieties and improved crop and seed production knowledge. Village Seed Banks were crucial to the production of quality seed of high-yielding chickpea varieties and for developing distribution channels to improve farmer access to quality seed although lack of seed storage was seen as a major issue by most farmers (Kumara Charyulu et al., 2018). Farmer to farmer seed exchange was responsible for 90% of adoption. Similarly, to the Ethiopian experience, the public sector success has stimulated interest by private sector to become involved in chickpea seed production (GLDC Annual Report, 2018, 2019).

One of the main drivers of adoption of improved chickpea varieties and the subsequent seed systems development was a strong export market. Of note, the Government of Myanmar has a flexible approach to supporting diverse methods of seed production which facilitates production and distribution. In addition, it is also supportive of export crops such as chickpea.

### Cowpea in Nigeria

Cowpea breeding efforts at the International Centre for Tropical Agriculture (IITA) began in early 1970s, focusing mainly on grain yield and pyramiding genes for individual disease and insect resistance. In the early 1980s, focus shifted to cowpea improvement for multiple disease and pest insect resistance, with acceptable seed quality traits (white and brown rough seed coat, nutritional quality and short cooking time). In the 1990s to early 2000s, the focus shifted again to the development of varieties with different maturity periods, growth habit, and suitability for intensive cropping systems as well as to *Striga gesnerioides* and *Alectra vogelii* and multiple disease and insect resistance (Singh et al., 1997). Of note, IT84S-2246-4, with combined resistance to *striga* and *alectra*, aphids, bruchids, thrips and a variety of diseases was developed and released to Nigerian farmers. From 2007, IITA through the Tropical Legumes projects (TL II Phase I and Phase II, TL III) worked closely with partners in Nigeria to build on progress from previous work. Activities were initiated on the identification of additional sources of resistance to drought and heat as well as previously mentioned biotic stresses by exploiting the germplasm collections (Boukar et al., 2018).

Through these efforts, improved, high yielding cowpea varieties with different maturity periods, multiple resistances to biotic stresses and drought tolerant were promoted resulting in the release of over 20 improved varieties in Nigeria since the early 1980s. Access to seed and information on improved varieties was fostered. Forty-two percent of the farmers have adopted improved varieties resulting into 26% increase in yields, 14% increase in production costs, 61% increase in net returns per hectare and 5% reduction in poverty incidence, equivalent to 930,000 people moving out of poverty (GLDC Annual report, 2019; Manda et al., 2019, 2020).

The partnerships between IITA, the Institute for Agricultural Research and the University of Agriculture, Makurdi were important for the release of improved cowpea varieties as well as for facilitating smallholder farmers’ access to high quality seed to enhance uptake of the improved varieties. To accelerate cowpea seed production and delivery to smallholder farmers, diverse strategies were used. One of the interventions was the establishment of multi-stakeholder platforms consisting of both public and private partners. These platforms provided linkages among cowpea value chain actors, provided awareness for training and skill enhancement, and enhanced efficiency and effectiveness in technology delivery, development and use. In addition, the platforms served as a critical channel through which farmers conveyed their preferences for traits and other varietal and seed attributes. A wide range of partners were engaged in seed production and dissemination of cowpea across the country. An integrated system was adopted where various actors were engaged in producing different seed classes. (TL III Final Project Report, 2019).

Investment in small seed packets for distribution to farmers raised awareness of as well as promoted improved varieties. Farmers were willing to buy seed of profitable new varieties that met market demands. The Government of Nigeria reclassified cowpea as a priority crop resulting in a nation-wide increase in consumption of cowpea further fostering demand.

Scaling-up outputs and outcomes from TL II through TL III achieved more impact and built sustainable seed systems for cowpea in Nigeria. Strengthened seed systems were conducive to increasing the involvement of private seed companies in cowpea which increased from 50 in 2017 to 314 in 2018. Improved grain quality was well-received by both local and export markets and good country-wide market access developed. Nigerian seed laws were revised to allow companies to produce foundation seed for certified seed production. The National Seed Regulation body (NASC) supported seed certification and quality control while the Economic Community of West African States (ECOWAS) supported the movement and sale of cowpea seed between countries.

### Tropical grain legumes in Nigeria, Tanzania and Uganda

To boost legume seed production and delivery to smallholder farmers, a multi-pronged strategy was used consisting of building partnerships between farmers, seed companies, governmental organizations and extension workers; training seed producers and marketers in technology and best practices through participatory varietal selection and on-farm demonstrations and mobile app-based advisories (TL III Final Project Report, 2019). The use of multi-stakeholder platforms (MSPs) to produce and deliver early generation seed (EGS), certified and QDS seed has been a successful approach. The established and strengthened MSPs were instrumental in producing and making seed available to smallholder producers at affordable cost (Akpo et al., 2020). The technical capacities of farmer seed producer groups were built for quality seed production, and the groups were contracted by public or private seed enterprises to produce foundation or certified seeds, at agreed buy-back prices. The challenges of quality and quantities of grain demanded by off-takers were resolved through discussions thereby stimulating sustainable demand and incentivizing farmers to procure high quality seed of improved varieties. The case studies highlighted above are part of the larger success stories of various models that worked and have remained as strong examples worth scaling out.

The Tropical Legumes (TL) Projects funded by the Bill and Melinda Gates Foundation together with precursor and complementary projects facilitated the development of 304 nutrient dense, climate-smart, farmer- and market-preferred varieties and the production of 397,050 t of certified and quality declared seeds (QDS) (groundnut in Tanzania – ICRISAT; cowpea in Nigeria – IITA; and common bean in Uganda – CIAT). TL III helped to scale out previous projects and seed was planted on about 4.4 million ha by more than 22 million smallholder farmers in the 15 target countries and beyond, producing about 4.9 million t of grain worth US$2.6 billion. In Tanzania, the groundnut area increased from 400–1.6 million ha and yields increased from 0.6–1.2 t/ha; in Nigeria, yields of cowpea increased from <0.5 to 1.1 t/ha by 2018

ICRISAT, IITA and the International Centre for Tropical Agriculture (CIAT) formed partnerships with more than 100 public sector institutions during the course of the projects. The value of innovative partnerships linked to seed producer groups was demonstrated. Improved quality control for production of QDS was conducive to attracting private seed companies to produce grain legume seed. The importance of market preferences to fostering demand was also clarified.

Quality seed of improved varieties fed into the multi-pronged strategy building partnerships between farmers, seed companies, governmental organizations and extension workers. Capacity building of seed producers and marketers in technology and best practices through participatory varietal selection as well as on-farm demonstrations and mobile app-based advisories was integral to successful outcomes. Market incentives were created through 55 multi-stakeholder platforms. In the case of Tanzania, the government increased support for legume crops of less interest to the private sector and established the Agricultural Seed Agency (ASA) to support multiplication of early generation seed for foundation seed. The Tanzania Official Seed Certification Institute (TOSCI) support for the production of QDS was a key development, lowering the cost of seed without compromising quality (15–30% cheaper than certified seed – Sperling et al., 2017).

## Lessons learnt and enabling factors

All four case studies highlighted at least some of the following measurable outcomes: increased adoption of improved varieties; increased area planted; increased productivity; increased income to farmers; improved market access and growth; increased welfare; and significant national economic benefits.

Investment in small seed packs of 2, 5 and 10 Kgs that farmers could afford was critical to creating awareness and promoting improved varieties in several case studies. The development of strong, long-term partnerships as well as capacity building was important in all cases. Strong partnerships also enabled the formation of multi-stakeholder platforms such as the EthioPEA Alliance in Ethiopia which was the foundation of the chickpea value chain. Strengthened seed systems attracted the interest of the private sector in Ethiopia, Nigeria and Myanmar. Such partnerships are crucial to scaling up outputs and outcomes for sustainable seed systems.

Adoption of innovations requires not just the right technology but also the conducive, enabling environment in which the technology is embedded (Sumberg, 2005). Orr (2018) identified three key factors needed to enable successful adoption: functional institutions, demanding markets and conducive policies. In all four case studies, functional institutions included not only the partnerships directly involved in producing, distributing and marketing grain legume seed but also other key institutions such as national governments, seed regulation bodies, the Ethiopian commodity exchange and the regional organization ECOWAS in the case of Nigeria. In all four cases, the existence and growth of local, national and export markets were important factors. Local markets were accessible and demand from both local and export markets drove increased production. For both studies on chickpea in Ethiopia and Myanmar, export markets were important drivers for adoption and investment in seed systems although models differed. ECOWAS supported the sale of cowpea seed between countries in West Africa. Finally, in all four cases, national seed policies were more or less conducive to promoting grain legume production and utilization as well as trade. In Ethiopia, the government included chickpea in its 5-year growth strategy and invested in both seed systems and market development; in Nigeria, the government reclassified cowpea as a priority crop resulting in a nation-wide increase in consumption of cowpea further fostering demand; while in Myanmar, the government supported diverse methods of seed production and promoted chickpea as an export crop.

It is also fortunate that predictable funding largely through bilateral projects was available to support seed systems activities especially from the Bill and Melinda Gates Foundation (BMGF) which funded Tropical Legumes projects I, II and III and has recently funded the AVISA project. However there is still a need for improved integration of donor-funded seed systems projects to enable the development of coherent national seed strategies (Tripp, 2000) while keeping an option open for multiple seed system models operating simultaneously, as evident in the case of Myanmar.

Although significant adoption of improved varieties of legume crops in Africa has been reported due to efforts in seed systems projects, the overall use remains low. Although interest by the private sector is growing, further expansion is compounded by the botanical challenges of the crops besides limited product information, including the technical and market performance of new varieties, a dearth of knowledge of the size and scale of the business opportunity, limited access to early generation seed (EGS) and obscurity about the licensing and regulatory environment. The BMGF’s investment in AVISA consolidates the gains made and lessons learnt from the Tropical Legumes Projects for better scale and efficiency. AVISA will address the private sector constraints by enabling the establishment of a robust system that (i) increases the quantity and quality of performance data substantiating varietal superiority; (ii) boosts the availability of EGS seed by strengthening the technical and business acumen of the public EGS systems through technical, management and business capacity building; (iii) establishes a clear path and handover process from the research system to the private sector; and (iv) enables private sector multipliers to seize opportunities to capitalize on the commercialization of these crops. The project is committed to linking with/aligning around new (some pending) institutional seed systems initiatives of BMGF, USAID and AGRA in these and other AVISA target geographies to achieve donor coherence and potentially greater outcomes than would be possible through independent efforts.

AVISA is also committed to gender equity as a guiding principle, considering the critical role women play in choosing legume varieties and seed sources. Women seed entrepreneurs and women-led seed companies will garner special attention for capacity development in seed business and technical areas. To ensure women farmers have equal access to improved seed, gender-responsive demand will be generated through linkages and innovative public-private partnerships and platforms with actors along the commodity value chains and through demonstrations that directly expose women farmers and other value chain actors to the material.

In summary, all four case studies created an enabling environment for successful innovations in grain legume seed systems. These case studies clearly show that identifying market demand correctly, working with innovative institutions/models to overcome constraints in production and delivery of improved seed and the existence of conducive policies to support seed systems are integral to their success. This is reinforced by a number of the principles identified by Ojiewo et al. (2018) for mainstreaming functional legume seed systems in Eastern Africa (Box 1).

## Future research needs

The analysis of these four case studies also identified a number of areas that require further research. Firstly, there is a need to better understand farmer demand for quality seed (Almekinders et al., 2019). Methodologies should be developed to capture the variable real-life situations of farmers to provide the metrics to enable seed actors and policymakers to make informed decisions. This should further enhance the development, uptake and impact of improved seeds to the benefit of smallholders. Secondly, there is a need to improve understanding of why and under what circumstances farmers decide to adopt improved varieties i.e. the drivers of adoption (Glover et al., 2019). In Ethiopia, adoption of improved chickpea varieties was driven more by profits and disease tolerance than by higher yields (Michler et al., 2018; Verkaart et al., 2017) however less is known about the drivers in the other three case studies. Thirdly, there is a need to better understand the complexities of market preferences (Waldmann et al., 2016). In Ethiopia and Myanmar, the rapid development of export markets was at least partly due to meeting market preferences, but less is known about local and national preferences. Finally, there is a need to better understand the key factors contributing to the establishment of sustainable seed systems as well as the critical bottlenecks that must be overcome for a strong structure, function and conductance of seed businesses. Although the role of private seed sector in legume seed systems in sub-Saharan Africa and South Asia is limited, the above cases clearly show that public sector success in achieving higher rates of variety adoption has generated interest from the private sector. The drivers that enabled the private sector to engage in legume seed systems also have to be studied and documented for spilling over the successful models.

## Concluding remarks

In spite of the successes shown by these four case studies in the adoption and spread of improved varieties and establishment of viable seed systems resulting in notable increases in productivity, such results can be patchy and restricted to intervention areas where projects are implemented. There is a need for further efforts to exploit available opportunities for achieving larger impacts by enhancing productivity and production of grain legumes (Ojiewo et al., 2018; Sperling et al., 2017). There is great value in learning from previous projects and spilling over successes where relevant. It is also clear that both the formal and informal seed systems are important for grain legumes. Both seed systems should be built on strategically and systematically.

It is somewhat ironic that although the Sustainable Development Goal 2 is to ‘End hunger, achieve food security and improved nutrition and promote sustainable agriculture’, insufficient attention has been paid to the important role of nutritious grain legumes in this objective. Governments, funders and the international community need to put much more effort into mainstreaming grain legumes in production systems to realize this critical goal.
